# Speed and accuracy of dyslexic versus typical word recognition: an eye-movement investigation

**DOI:** 10.3389/fpsyg.2014.01129

**Published:** 2014-10-09

**Authors:** Richard Kunert, Christoph Scheepers

**Affiliations:** ^1^Neurobiology of Language, Max Planck Institut für PsycholinguistikNijmegen, Netherlands; ^2^Neurobiology of Language, Donders Institute for Brain, Cognition and Behaviour, Radboud University NijmegenNijmegen, Netherlands; ^3^Institute of Neuroscience and Psychology, University of GlasgowGlasgow, Scotland

**Keywords:** visual word recognition, dyslexia, speed-accuracy trade-off, consistency, phonology

## Abstract

Developmental dyslexia is often characterized by a dual deficit in both word recognition accuracy and general processing speed. While previous research into dyslexic word recognition may have suffered from speed-accuracy trade-off, the present study employed a novel eye-tracking task that is less prone to such confounds. Participants (10 dyslexics and 12 controls) were asked to look at real word stimuli, and to ignore simultaneously presented non-word stimuli, while their eye-movements were recorded. Improvements in word recognition accuracy over time were modeled in terms of a continuous non-linear function. The words' rhyme consistency and the non-words' lexicality (unpronounceable, pronounceable, pseudohomophone) were manipulated within-subjects. Speed-related measures derived from the model fits confirmed generally slower processing in dyslexics, and showed a rhyme consistency effect in both dyslexics and controls. In terms of overall error rate, dyslexics (but not controls) performed less accurately on rhyme-inconsistent words, suggesting a representational deficit for such words in dyslexics. Interestingly, neither group showed a pseudohomophone effect in speed or accuracy, which might call the task-independent pervasiveness of this effect into question. The present results illustrate the importance of distinguishing between speed- vs. accuracy-related effects for our understanding of dyslexic word recognition.

## Introduction

It is estimated that developmental dyslexia is a reading impairment affecting between 5 and 17.5% of the population (Shaywitz, 1998; Vellutino et al., 2004). The underlying cognitive deficit is often investigated using a visual lexical decision task (LDT) in which a participant is asked to judge whether a given letter string is a word or not by pressing one of two buttons (e.g., Bergmann and Wimmer, 2008). The nature of the deficit has remained controversial with proposals relating dyslexia to attentional (Hari and Renvall, 2001; Bosse et al., 2007), visual (Stein, 2003), and auditory deficits (Tallal, 1980) among others. In the presented study we focused on the two most widely reported problems in dyslexic word recognition—a phonological core deficit combined with a general deficit in processing speed (Vellutino et al., 2004; Swanson and Hsieh, 2009). Our paradigm allowed us to tap into both issues simultaneously within the same task.

Evidence for a phonological core deficit in dyslexic readers is based on a variety of experimental paradigms (*cf*. Vellutino et al., 2004), especially the LDT (e.g., Bergmann and Wimmer, 2008; Lavidor, 2011; Zeguers et al., 2011; Mahé et al., 2012). The latter targets word recognition, which Vellutino et al. (2004, p. 6) identified as the “most ubiquitous cause of difficulties in learning to read.” However, as we shall discuss in detail later, the lexical decision paradigm is poor at distinguishing between a dyslexic *speed* deficit (which would suggest a delay in accessing word representations) and a dyslexic *accuracy* deficit (which would suggest a deficit in the quality of word representations)^1^. This is because participants can trade off speed for accuracy in such tasks. For example, let's assume that dyslexic readers have task-appropriate phonological representations, but that they require more time to access those representations. Since timing of responses is under their control, they could decide to respond quickly (at the expense of making more errors), particularly when presented with “difficult” stimuli such as infrequent words or “word-like” non-words. This would result in an apparent deficit in the quality of phonological representations while in fact, speed of access to those representations is impaired.

The need to distinguish between speed and accuracy is particularly pronounced in dyslexia research because either speed or accuracy deficits have been claimed to be at the heart of dyslexic impairment (e.g., Wolf and Bowers, 1999; Vellutino et al., 2004). Using a novel two-alternative forced choice LDT which avoids speed vs. accuracy trade-offs (SAT) we investigated how different linguistic variables affect the speed and accuracy of word/non-word recognition in both dyslexic and control participants. On each trial, our task presented both a real word target and a non-word distractor simultaneously. Participants were asked to look at the real word and ignore the non-word while their eye-movements were recorded. This allowed us to track both the speed of real word identification as well as its asymptotic accuracy. Crucial to our task were sensitive manipulations of real word targets and non-word distractors, which we will explain further below.

Given our interest in the speed and accuracy of phonological processing in dyslexic readers, the present study primarily manipulated phonology-related variables. Coltheart et al.'s (2001) Dual Route Cascaded (DRC) model can be used to clarify the task demands as well as the proposed dyslexic deficit. The DRC model assumes two independent processing routes to correct word identification in reading. Firstly, a *lexical* route whereby letter-strings activate orthographic representations of whole words. Secondly, a *non-lexical* route in which phonemes are activated incrementally (letter-by-letter) on the basis of learnt rules; the completed string of phonemes will eventually give access to the word, after checking its lexicality via a search in the mental lexicon. The hypothesized phonological core deficit in dyslexic readers would constitute an impairment of the grapheme-phoneme conversion rule system necessary for the second, non-lexical route to word recognition. This rule system is crucial when trying to pronounce novel words or non-words, for example. In our task, we crossed different types of words and non-words of varying difficulty, targeting in particular the non-lexical route to word recognition as the hypothesized locus of dyslexic impairment.

The present manipulation of non-words comprised three different kinds of distractors. Firstly, unpronounceable (UP) non-words (e.g., *necltb*) which are fairly easy to identify as non-words because they contain illegal strings of graphemes that are detectable even during pre-lexical analysis (an initial orthographic analysis in the DRC model). Secondly, pronounceable (P) non-words (e.g., *stoint*), which should be more difficult to reject in a LDT (*cf*. Stone and Van Orden, 1993) because the reader would have to use the non-lexical route (assumed to be impaired in dyslexics) in order to convert graphemes into a string of phonemes and then check whether a corresponding entry exists in the lexicon. Alternatively, these letter strings could be directly compared to entries in the orthographic lexicon. Thirdly, so-called *pseudohomophones* (PH) (e.g., *lepht*) which, when appropriate grapheme-phoneme conversion rules are applied, sound like existing real words, but they have a spelling that does not correspond to any lexical entry. This third type of non-word is assumed to be particularly difficult to reject in a LDT. This is because PH have phonological representations that are identical to (or at least strongly overlapping with) those of existing words in the mental lexicon. Therefore, their rejection must be solely based on the activation level of the orthographic lexicon (e.g., Ziegler et al., 2001). Frost's (1998) review of PH effects indicates that this type of non-word is indeed quite difficult to reject by unimpaired readers in a variety of tasks, e.g., letter-search tasks, semantic priming, and of course the LDT. For dyslexic readers, given that they show problems with pronouncing unfamiliar letter strings (Rack et al., 1992; Gallagher et al., 1996; see Atchley et al., 2003), PH like *lepht* may not be any more difficult to reject than P non-words such as *stoint*.

Apart from non-word reading, the hypothesized dyslexic problem of poor phoneme-grapheme conversion should also be visible when judging the lexicality of real words whose spelling is pronounced in more or less unusual ways. Such pronunciation problems were manipulated at the level of the word's rhyme unit in terms of *consistency* (Ziegler et al., 1997). Consistency, in its broadest sense, refers to “the extent to which spelling and sound co-vary in a predictable way” (Bosman et al., 2006, p. 272). For example, when judging the word *push* the conversion of the written rhyme -*ush* into its phonological code is made more difficult by words like *hush* and *rush*. Therefore, rhymes like -*ush* are said to be inconsistent from spelling to sound (*feedforward* inconsistent). On the other hand, words ending on –*ing*, e.g., *king*, are always pronounced as /-*iG*/ and are therefore said to be feedforward consistent. Interestingly, previous research on feedforward consistency effects in both adult and child dyslexic readers revealed conflicting results, which in a meta-analysis (Metsala et al., 1998) turned out to suggest no systematic differences between dyslexics and reading-age controls.

However, establishing a grapheme-phoneme rule system for reading might also be affected by the ease with which one can infer the spelling of a spoken word. Consider the rules “-*ing* ↔ /-*iG*/” and “-*in* → /-*In*/.” The first is a bidirectional rule: all words ending -*ing* are pronounced /-*iG*/ and vice versa. However, the second rule is unidirectional because /-*In*/ is written differently in *bin* and *inn*. Therefore, the first rule is applied more often (reading and writing) than the second one (reading only). This might lead to a better representation of rules of the first kind (Stone et al., 1997). The difference between these two kinds of rhymes will be referred to as *feedback* consistency^2^. The rhyme /-*In*/ is feedback inconsistent as it can be written in multiple ways. It is notable that previous research often failed to control for feed *back* consistency when investigating the effect of feed *forward* consistency as in the studies covered by Metsala et al. (1998). Thus, supposedly consistent words might have been feed *back* inconsistent (see Stone et al., 1997; Ziegler et al., 1997). This could explain a similar performance in both the consistent and the inconsistent conditions. Since then, to our knowledge, no study investigating consistency effects on word recognition in adult dyslexic readers has been published (but see Davies and Weekes, 2005; Bosman et al., 2006 for some findings on dyslexia in children). Given feedforward and feedback influences on lexical decision performance (Stone et al., 1997; Davies and Weekes, 2005), we manipulated both dimensions in our real-word targets.

Apart from the phonological core deficit, dyslexics are also known to be generally slower than normal readers across tasks, including processing speed tests of intelligence (Hatcher et al., 2002; Meyler and Breznitz, 2003; Miller-Shaul, 2005; Ingesson, 2006; Stoodley and Stein, 2006; Laasonen et al., 2009) and other non-linguistic tests (Stoodley and Stein, 2006; Laasonen et al., 2009; Swanson and Hsieh, 2009; but see Snowling, 2008). As a consequence, Wolf and Bowers (1999) suggested that two more or less independent deficits underlie dyslexia: a phonological core deficit and a general processing speed deficit.

Given the potential dual nature of dyslexic impairment, it is surprising why no research so far has studied dyslexic word recognition using an experimental paradigm that is less prone to speed-accuracy trade-offs than classical lexical decision or naming tasks. Indeed, the proposed general processing speed deficit in dyslexics could at least partly be due to a deficit in the phonological representation of words: because of the latter, dyslexic readers may adjust their lexical decision strategy to keep overall error rate at an acceptable level (by responding more slowly in general). Conversely, what appears to be a deficit in phonological representation (as reflected in reduced response accuracy) could to some extent be the result of a strategy whereby dyslexics try to compensate for their general slowness in responding (i.e., accepting more errors for a gain in speed). The fact that classical tasks are prone to such speed-accuracy trade-offs makes it difficult to infer whether dyslexic word recognition is best characterized by speed deficits, representational (i.e., accuracy) deficits, or both (as the dual deficit hypothesis suggests).

**Figure 1:**
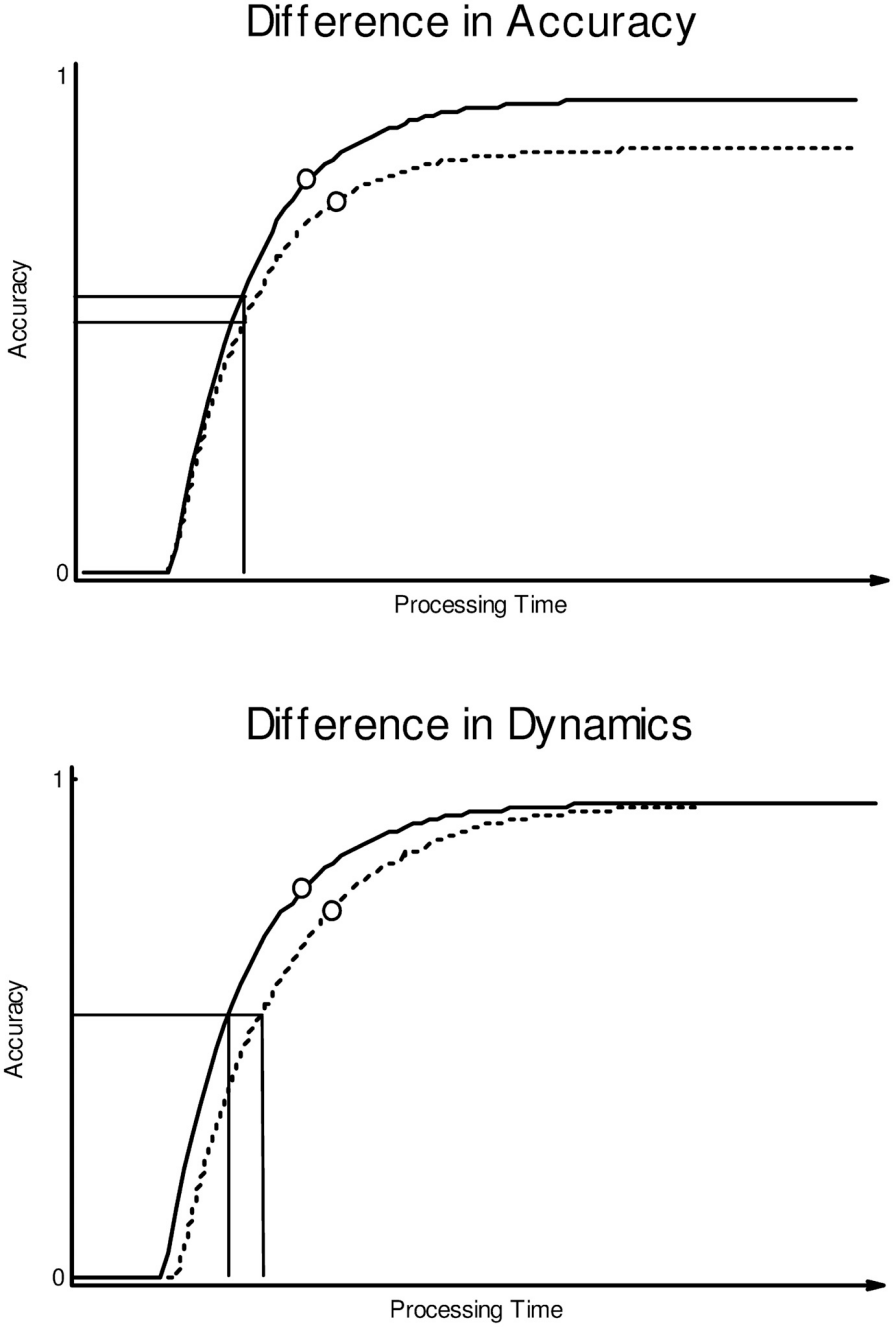
**Accuracy (y-axis) as a function of time (x-axis) for two hypothetical conditions (solid vs. dotted curves)**. The **top panel** illustrates a cross-condition difference in accuracy, the **bottom panel** shows a difference in processing dynamics. Horizontal and vertical lines map the 50% asymptotic accuracy level per condition onto corresponding temporal coordinates. The open circles on the curves indicate hypothetical average responses (in time × accuracy space) from a classical RT experiment (see text for further detail).

The problem of speed-accuracy trade-off in classical reaction time tasks has long been recognized in the literature, leading to the development of tasks such as the response signal paradigm (e.g., Reed, 1973; Wickelgren, 1977; Ratcliff, 1978; Dosher, 1979, 1982; McElree and Dosher, 1989; McElree and Carrasco, 1999). In this paradigm, participants are required to make a judgment on a stimulus (e.g., a letter string) as soon as an auditory cue is presented. The timing of the auditory cue varies continuously and is not under the control of the participant (participants are usually trained to respond to the cue with minimum delay). When judgment accuracy is analyzed as a continuous function of cueing-time, participants typically show zero accuracy (chance-level performance) very early on; over later time periods (i.e., after more information processing on the stimulus has taken place), accuracy gradually rises toward an asymptote in a non-linear fashion, as illustrated in Figure 1.

When fit by an appropriate non-linear function, cross-condition differences in asymptote (Figure 1, top) would indicate a difference in overall *accuracy* independent of timing. In the context of word recognition, such a pattern might arise, for example, when some of the words in the lower-asymptote condition are simply unknown to participants. A different situation is illustrated in Figure 1, bottom: The two conditions rise to the same asymptote (no difference in overall accuracy) but differ in the length of the initial zero-accuracy period and/or the rate at which accuracy increases over time; this would indicate a difference in *processing dynamics* independent of accuracy (e.g., words in condition B are recognized more slowly than in condition A, but equally likely to be recognized given sufficient time).

The response signal paradigm makes it possible to “map out” improvements in accuracy over time, virtually eliminating the possibility that participants adjust their response criteria to trade off speed against accuracy across conditions. This contrasts with classical reaction time tasks where participants are free to decide when and at what confidence level they provide their judgments (and where there is no guarantee that the relevant response criteria remain consistent across conditions). As an illustration, the open circles on the curves in Figure 1 represent hypothetical average responses from a classical reaction time experiment; the first condition is assumed to elicit consistently faster and more accurate responses than the second condition^3^. As can be seen, these classical RT differences are compatible with an underlying difference in accuracy only (Figure 1, top), a difference in processing dynamics only (Figure 1, bottom), or indeed a combined speed and accuracy difference (not shown in the figure). This goes to show that results from traditional reaction time experiments may remain ambiguous as to the true generative process behind cross-condition differences in processing speed and accuracy. The present study uses a novel lexical decision paradigm (close in spirit to the response signal paradigm) to obtain a better understanding of speed vs. accuracy deficits in dyslexic word recognition.

Specifically, the present study uses a two-alternative forced choice LDT based on eye-tracking which was first introduced by Scheepers and Shillcock (2004). In this task, two letter strings are presented simultaneously on screen for 3 s. One of the two strings per display is an existing word of the English language (the *target*) and the other one is a non-word matched in length and bigram frequency with the real word (the *distractor*). The positioning of the target and distractor strings is counterbalanced across trials. The participants' task is to only ever look at what they think is the target word in the display, and to ignore the distractor non-word as much as possible. Throughout this task, participants' eye-movements are continuously monitored. The latter makes it possible to map out how word recognition accuracy (more looks to the target, fewer looks to the distractor) gradually improves over time. Importantly, the relatively long presentation period of 3 s (which is about four to five times longer than commonly reported lexical decision times) ensures that participants are close to achieving asymptotic performance toward the end of the trial.

Compared to classical reaction time tasks, the present task is less prone to speed-accuracy trade-off because participants provide a continuous response (gradually “homing-in” on the target) rather than being required to make a single judgment. Moreover, it allows us to explicitly model improvements in word recognition over time, akin to the kind of analyses that are usually performed on data from the response signal paradigm. However, the present task does not require any training of participants, nor does it require paying attention to an auditory response cue. Therefore, our task combines the easy application of classical LDTs with the response signal task's ability to eliminate speed-accuracy trade-offs in responding. The latter is especially important when testing populations with hypothesized processing speed differences.

The experiment reported below compared the lexical decision performance of high functioning adult dyslexic readers with that of non-dyslexic control participants. Given the hypothesized lower processing speed of dyslexic readers, we expected dyslexic readers to perform more slowly overall than normal readers. Two factors were manipulated in our stimulus displays: the rhyme consistency of the target word and the “wordness” of the distractor non-word it was combined with.

The target word in each display was either both feedforward and feedback *consistent*, or it was *inconsistent* in both respects. Therefore, contrasting with previous investigations (*cf*. Metsala et al., 1998), rhyme consistency was manipulated simultaneously on both dimensions (feedforward *and* feedback). We expected an overall effect of consistency across both groups of participants because inconsistent words are more difficult to process for the non-lexical route in Coltheart et al.'s (2001) DRC model. That is, rhyme-inconsistent words were expected to be recognized more slowly (and possibly less accurately) than rhyme-consistent words. Moreover, we expected that this effect should be particularly pronounced in dyslexic readers, whose non-lexical route is thought to be impaired, as compared to normal readers.

The target words were combined with three different types of non-word distractors: unpronounceable distractors (UP for short), pronounceable distractors (P) and pseudohomophone distractors (PH). For our non-dyslexic control participants, we predicted that they should find it harder to perform the task the more “word-like” the distractor would appear to them (resulting in more competition or interference from the distractor). Thus, firstly, we expected that they should identify the target word most quickly in combination with a UP distractor which can be rejected based on a pre-lexical analysis of unusual letter combinations (initial orthographic analysis in terms of the DRC model). Secondly, they should be slower in combination with a P distractor which can only be rejected based on an absent entry in the lexicon. Thirdly, they should react least quickly in combination with a PH distractor which might lead to conflict between an entry in the phonological lexicon and an absent entry in the orthographic lexicon. Thus, the expected pattern can be summarized as UP < P < PH. For dyslexic readers on the other hand, given their hypothesized phonological decoding deficits, we expected distractor-related interference effects to be less pronounced. Presumably there is no clear difference in performance between the P and PH distractor conditions (P ≈ PH) since an impaired non-lexical route is less likely to lead to a phonological representation of PH non-words. Therefore, such an absent representation cannot interfere with a word judgment based on orthographic lexicon entries. Note that UP non-words are also likely to violate orthographic conventions, probably allowing both normal and dyslexic readers to reject them with relative ease.

## Materials and methods

### Design

The experiment employed a 2 (participant group) × 2 (rhyme consistency of the target) × 3 (type of non-word distractor) mixed design. Participant group (dyslexia vs. control) was between-subjects and the remaining factors were within-subjects.

### Participants

Twenty two University of Glasgow undergraduates took part in the study. They were all native English speakers and had normal or corrected-to-normal vision. Participants were either paid £6 or course credits for their participation. The study was approved by the Faculty of Mathematical and Information Sciences ethics committee at Glasgow University and informed consent was obtained from all subjects. The *dyslexic* group consisted of 10 participants who had been independently diagnosed as dyslexic, as recognized by the Disability Support Service at Glasgow University. The *control* group comprised 12 age- and gender-matched participants without any known history of dyslexia. Before the experiment proper started, all participants were prescreened for risk of dyslexia using the Lucid Adult Dyslexia Screener (LADS, Singleton et al., 2009). This pre-test confirmed that each of the 10 participants in the dyslexic group—but none of the 12 participants in the *control* group—showed at least “borderline risk” of dyslexia. Table 1 provides further details of the pre-screening results (including LADS sub-scales) and person-specific variables per group. Independent-measures *t*-tests confirmed that the two groups differed reliably only on the two language-related sub-scales of the LADS (*word recognition* and *word construction*), as well as on the overall LADS test score. There were no significant group differences in the *non-verbal reasoning* (general intelligence) and *working memory* sub-scales of the LADS, and nor were there any substantial differences in age or gender distribution.

**Table 1:** **Participant details shown as means (ranges in brackets) per group**.

**Group**	***N* ♂**	**Age**	**LADS test scores**				
			**GR**	**Wrec**	**Wcon**	**WM**	**LADS**
Dyslexic	4/10	21.5 (18–32)	4.4 (3–5)	4.2 (3–8)	6.1 (3–8)	2.0 (1–6)	12.3 (9–17)
Control	4/12	20.8 (18–31)	3.9 (3–5)	2.0 (1–3)	2.5 (2–3)	1.4 (1–3)	5.9 (4–9)
*P*	>0.7	>0.6	>0.1	<0.001	<0.001	>0.3	<0.001

*Age is given in years. LADS test scores are broken down by sub-scale; GR, general reasoning (scores ranging from 1 = “poor” to 5 = “excellent”); Wrec, word recognition; Wcon, word construction; WM, working memory (each with scores ranging from 1 = “excellent” to 9 = “poor”). The LADS sub-score in the rightmost column is a function of Wrec, Wcon, and WM. GR is combined with the LADS sub-score to determine a final “risk of dyslexia” classification (identical LADS sub-scores therefore do not necessarily lead to the same classification). The bottom row shows uncorrected p-values from 2-tailed between-group t-tests on each variable*.

### Materials

Three-hundred-sixty-six stimulus pairs were created, each consisting of a target word combined with a distractor non-word. The complete list of stimuli can be found in the Supplementary Material. The distractor non-words were always matched in length with the target words they were paired with (ranging from 3 to 8 characters across items). As will be explained below, two types of target words (rhyme-consistent vs. rhyme-inconsistent) were paired with three types of distractor non-words (UP, P, PH), giving six experimental conditions altogether: consistent-UP, consistent-P, consistent-PH, inconsistent-UP, inconsistent-P, and inconsistent-PH. Each participant was exposed to 61 items per condition.

The target words were either rhyme-*consistent* or rhyme-*inconsistent*, following the definition in Ziegler et al. (1997). They sampled all monosyllabic and monomorphemic words (*N* = 2694) contained in Kučera and Francis (1967) and determined their rhymes' dictionary pronunciations. A rhyme spelling with different pronunciations in different words was labeled *feedforward* inconsistent. A rhyme pronunciation with different possible spellings was labeled *feedback* inconsistent. For the present study, *consistent* target words always comprised a rhyme from Ziegler et al.'s (1997) database that was classified as both feedback and feedforward consistent.

The generation of inconsistent target word stimuli proceeded in four steps. First, all feedforward-inconsistent rhymes in Ziegler et al. (1997) were extracted. For example, the rhyme spelling “-*our*” is feedforward inconsistent because it has four possible pronunciations: /*or*/ (as in *four*), /*R*/ (as in *your*—considering American and Scottish variants of English), /*Ur*/ (as in *tour*) and /*Wr*/ (as in *flour*). Second, of these feedforward-inconsistent rhymes, only those grapheme pronunciations which were less frequent were chosen. For example, all words in which –*our* is pronounced /*Wr*/ have a combined frequency higher than the words whose rhyme spelling was pronounced in other ways. Therefore, words in which –*our* is pronounced /*Wr*/ were excluded. Third, all the remaining feedforward-inconsistent rhymes were checked for feedback-consistency, and only the feedback-inconsistent ones were retained. For example, the pronunciation /*Wr*/ (as in *flour*) can be written in only one-way, thus it is feedback consistent. Therefore, words in which –*our* is pronounced /*Wr*/ had an additional reason to be excluded from the list of inconsistent words. Fourth, all remaining rhymes' real words were searched in the BYU-BNC corpus of written British English (Davies, 2004) and included in the study. For example, all monosyllables ending in –*our* and pronounced /*or*/ (as in *four*) were looked up in the BYU-BNC and included.

The target words were paired with UP, P, or PH non-word distractors of the same length. We used the *WordGen* software (Duyck et al., 2004) to randomly generate UP and P distractors; the former (UP) were always without orthographic neighbors. PH distractors were derived by changing certain letter combinations in a third of the original target words (e.g., converting “*left*” into “*lepht*”). These changes were based on pronunciation intuitions of the first author. We will come back to this point in the discussion. PH distractors were never paired with the target words they were derived from. All non-words were monosyllabic, just as their target word counterparts.

Across items, the six experimental conditions (consistent-UP, consistent-P, consistent-PH, inconsistent-UP, inconsistent-P, and inconsistent-PH) were matched on a number of variables. On average, target words were of roughly the same length (all *p*s > 0.1 by independent-samples *t*-tests comparing each condition with each other)^4^, frequency (values from Davies, 2004; all *p*s > 0.1), log frequency (all *p*s > 0.1), and bigram frequency (Celex values from Baayen et al., 1993; all *p*s > 0.05). In the case of rhyme-inconsistent target words, we also made sure that there were no systematic differences in *fluency*, i.e., the proportion of word frequencies with the given grapheme and rhyme pronunciation out of all word frequencies with the given grapheme (values from Kučera and Francis, 1967; all *p*s > 0.05).

Within conditions, targets and distractors were matched in terms of bigram frequency (values from Celex; all *p*s > 0.05). Moreover, PH distractors were matched in terms of log base word frequency (values from Davies, 2004; all *p*s > 0.1).

### Procedure

Each participant first completed the LADS pre-screening (see Participants section) which took about 15–20 min. During the main experiment, eye movements were monitored using an SR-Research Eyelink II (SR Research LTD, Mississauga, Ontario, Canada) head-mounted eye tracker, which has a sampling rate of 500 Hz, a spatial resolution of 0.01° and a spatial accuracy of 0.1°. Although viewing was binocular, only the participant's dominant eye was tracked (as established via a simple parallax test). Each participant was seated about 70 cm from a 21 inch CRT display running at 85 Hz refresh rate with 1024 × 768 pixel resolution. A chin rest was used to keep viewing distance constant and to prevent strong head-movements during tracking.

At the beginning of the experiment proper, the eye-tracker was calibrated using the standard EyeLink calibration and validation procedures, in which the participant had to successively fixate nine dots in various positions on the screen. The experiment was divided into six blocks of 61 trials each. At the beginning of a new experimental block, participants were given a short break if required, and the eye-tracker calibration and validation procedures were repeated. Within each block, the experimenter could interrupt the procedures at any time to re-calibrate the eye-tracker if necessary (e.g., after a change in the participant's posture). The 366 experimental trials appeared in a random sequence, determined individually for each session. Every word non-word pair only appeared once in the experiment.

Each individual trial started with the presentation of a central fixation dot. The participant looked at it while the experimenter initiated a semi-automatic drift correction (taking less than 250 ms), followed by the presentation of the stimulus. The stimulus always consisted of a pair of (equally long) letter strings whose enclosing-rectangle midpoints were ca. 1.5 degrees of visual angle above and below the previously presented central fixation dot. The letter strings were presented in 28 pt Courier Bold font, presented in black on a light-gray background. In half of the trials (*N* = 183), the letter string at the top was the target word and the letter string below the non-word distractor, and vice versa in the other half of the trials. The participant's task was to “*only ever look at the word, and to ignore the non-word presented in each display*.” After exactly 3000 ms, the stimulus disappeared from the screen and the next trial was initiated after a 300 ms blank-screen period.

The positioning of the target vs. distractor strings was counterbalanced across two presentation lists. In each list, ca. half of the items per condition had the target word at the top, and the other half had the target word at the bottom. Across lists, relative positionings of targets and distractors were swapped on an item-by-item basis. Each list was seen by 50% of the participants per group. A typical experimental session (including breaks in-between) took about 75 min to complete.

### Data analysis

The eye-tracker provided continuous eye-fixation data per trial, whose bitmap coordinates were mapped onto regions of interest by means of color-coded bitmap templates. These templates coded the position of the target word and the distractor non-word in each display in terms of their rectangular perimeters. Whenever a fixation landed in one of those rectangles (expanded by about 15% in each dimension, as fixations often land slightly outwith the letter-string for inspection), it was classified as being on the target word or on the distractor non-word, respectively. Any other region was coded as whitespace.

**Figure 2:**
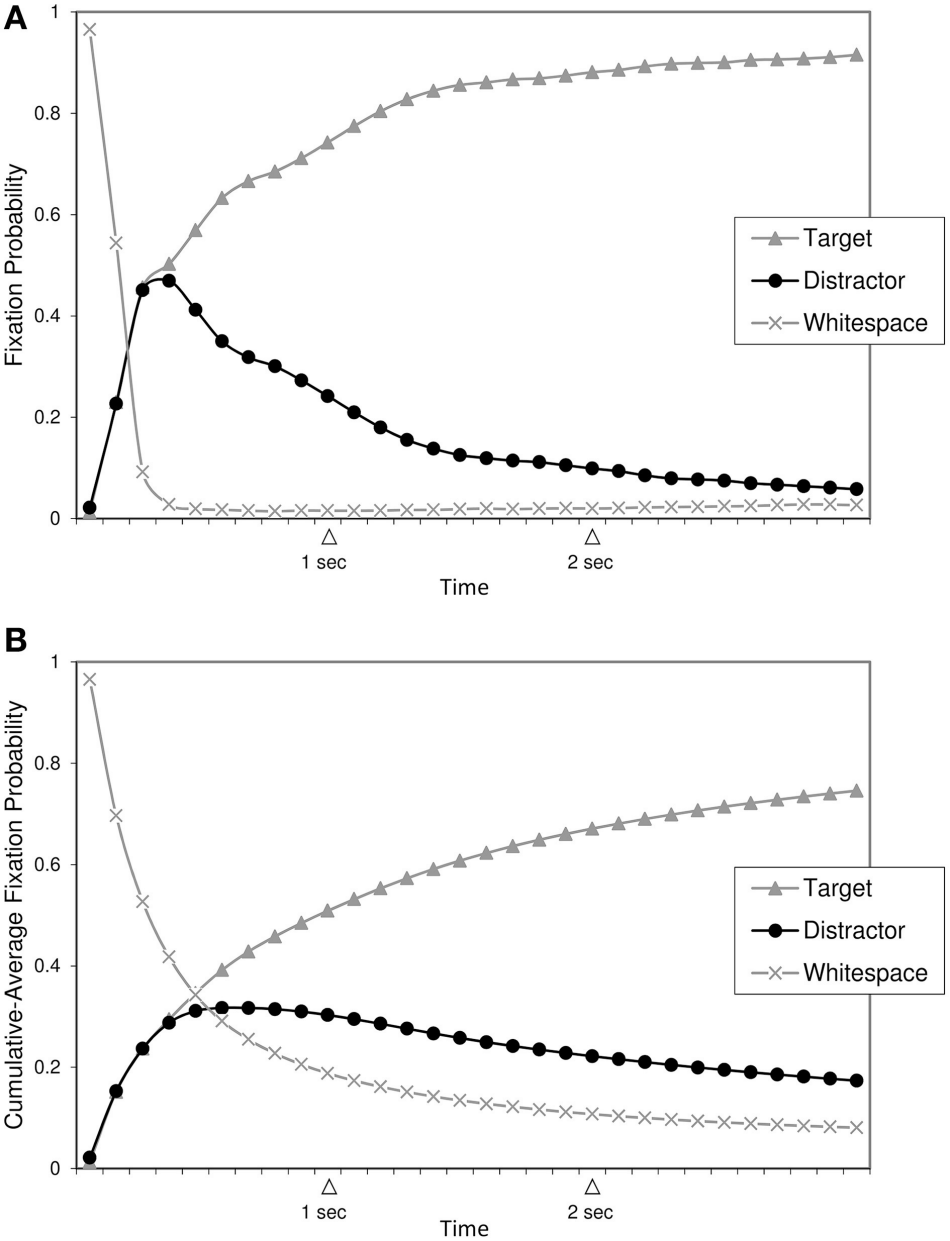
**(A)** Fixation probabilities per region over time. **(B)** Cumulative-average fixation probabilities per region over time. Regions of interest are the Target word, the Distractor non-word, and Whitespace (any other region).

From these data, we computed fixation probabilities over time, after reducing the temporal sampling rate to 10 Hz (one time sample every 100 ms). Grand averages of these data (for each region of interest, averaged across participants and conditions) are shown in Figure 2.

As can be seen in the top panel (Figure 2A), participants gradually “home in” on the target word as time progresses, that is, the proportion of fixations on the target word steadily increases at the expense of fixations on other regions. Note that within a time period of ca. 0–400 ms after stimulus onset, proportions of fixations on the whitespace region sharply drop from initially close to 100% (which is expected because the central fixation dot at the start of each trial is also part of the whitespace region) to nearly 0%; more interestingly, proportions of fixations on the two critical regions indicate no preference for the target word over the distractor non-word during this 400 ms period, suggesting that perceivers are still in the process of collecting information about the two letter strings without being able to make a lexical decision.

Toward the end of the 0–400 ms period, proportions of “incorrect” fixations on the non-word distractor have risen to a peak. From then on, the likelihood of fixating the distractor decreases monotonically in favor of more fixations on the target word. At the end of the trial (3000 ms after stimulus onset), lexical decision accuracy reaches its maximum of ca. 90% on average. Thus, performance on the present task appears to involve two markedly different time-intervals, separated by the point in time at which “incorrect” distractor fixations are at maximum likelihood: an “*initial uncertainty time*” during which perceivers seem unable to make a lexical decision, followed by a “*home*-*in time”* during which perceivers correctly discriminate between target and distractor with steadily increasing accuracy.

Figure 2B (bottom panel) shows the same information, but with the data being converted into so-called *cumulative averages* per time bin. For any given time bin, the cumulative average proportion of fixations on a region is defined as the average proportion of fixations on that region *up to and including* the given time bin. So, for an imaginary time series like *t*_0_ = 0.10, *t*_1_ = 0.30, *t*_2_ = 0.50, cumulative averages would amount to 0.10 at *t*_0_, to (0.10 + 0.30)/2 = 0.20 at *t*_1_, and to (0.10 + 0.30 + 0.50)/3 = 0.30 at *t*_2_, respectively. Cumulative averages are useful to smooth out short-term fluctuations and highlight global trends in time series data, which is why we will use cumulative averages for our curve-fitting analyses below^5^. However, it is important to bear in mind that cumulative averages have a slightly different interpretation compared to raw averages. While Figure 2A shows how likely it is that a given region is fixated *at a particular point in time*, Figure 2B shows how likely it is that the region is fixated *up to (and including) a particular point in time*. Also note that cumulative-average smoothing results in “flatter” curves overall, as well as in a temporal delay of the location of the peak (for probability of looks to the non-word distractor) when comparing Figure 2B with Figure 2A.

In the analyses that follow, we modeled cumulative average fixation probabilities on the distractor non-word (conceptually equivalent to cumulative average *error rates*) as a continuous function of time, after aggregating the data up to the participant × condition level. We explored a range of differently shaped peak-distribution functions (using *Systat TableCurve 2D*) and identified a four-parameter log-normal peak function as the best descriptor of both within- and between-condition variability in our data. The mathematical definition of this function is given in (Equation 1); PD(*t*) refers to the cumulative average probability of fixating the distractor non-word as a function of time (*t*).

(1) $$\text{PD}(t)=\lambda \;exp\left[\frac{-ln(2)}{ln{\left(\gamma \right)}^{2}}ln{\left(\frac{\left(t-\delta )(\gamma^{2}-1\right)}{\beta \gamma }+1\right)}^{2}\right]$$

**Figure 3:**
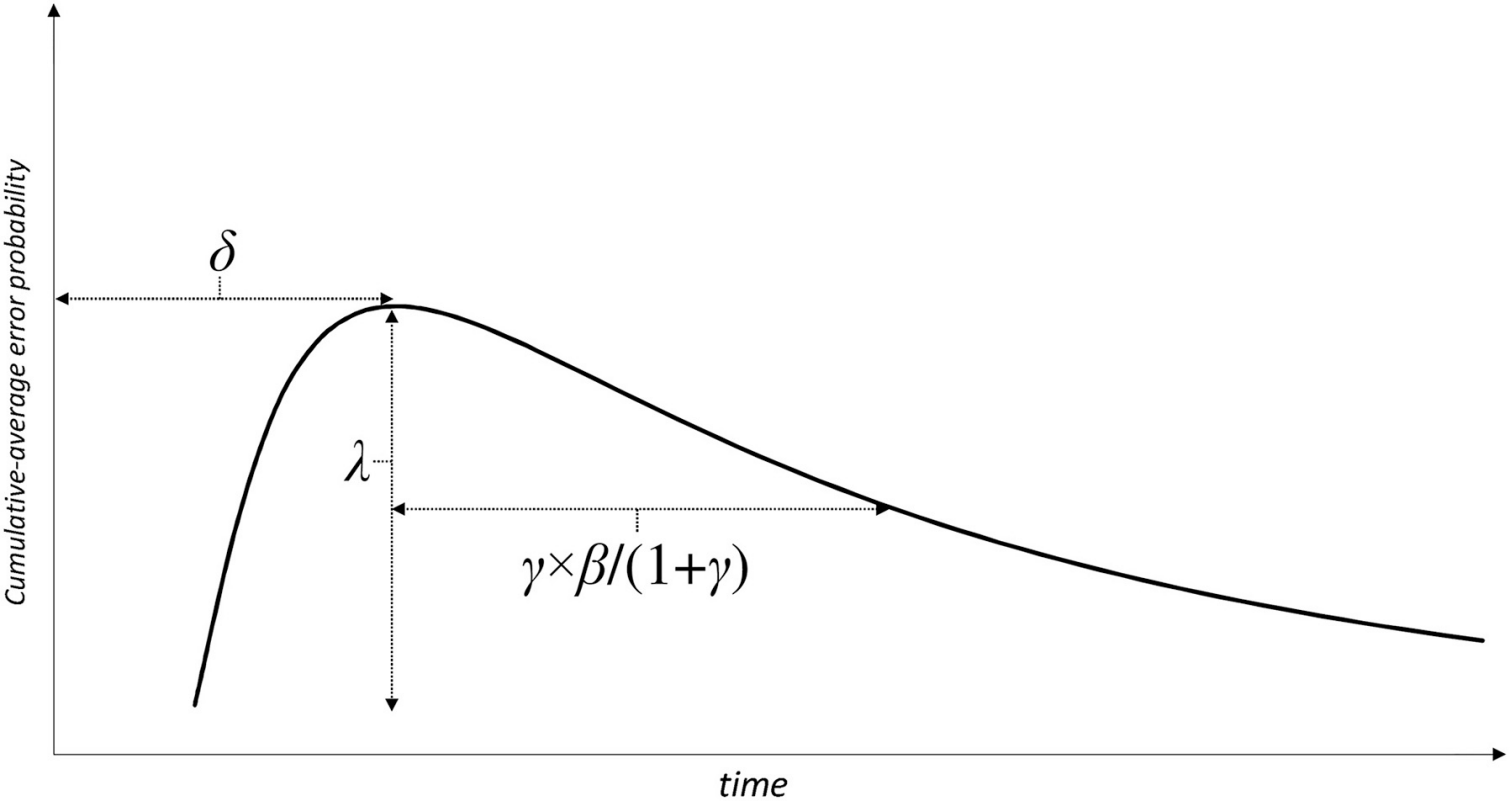
**Analysis measures derived from the log-normal peak function parameters**. δ indexes the peak location in time (henceforth called “initial uncertainty time” since proportions of looks to the target vs. distractor do not diverge up to this point, see Figure 2); the peak amplitude λ is a measure of overall error rate (complementary to accuracy; note that we are modeling proportions of looks to the distractor); the compound measure γ × β /(1 + γ) (henceforth called “home-in time”) captures the time that elapses between the peak location δ and the point in time where error rate has dropped to ½λ in the right tail of the distribution.

Across participants and conditions, the model obtained an average goodness of fit of *R*^2^_adj_ = 0.985 (Min. = 0.886, Max. = 0.998). There were no systematic differences in terms of goodness of fit between the dyslexia group and the control group. The function in (Equation 1) comprises four free-varying parameters: the peak amplitude (λ), the peak location in time (δ), the width of the distribution at half amplitude (β), and a symmetry parameter (γ) which controls the rate of decline from the peak in the right tail of the distribution. After fitting this function to each individual participant × condition dataset, we derived the following three measures of interest from the parameter estimates (illustrated in Figure 3) which formally capture the descriptive observations made earlier.

The peak location in time (δ) marks the endpoint of what we termed the *initial uncertainty time*, during which perceivers do not visually discriminate between the target word and the distractor non-word. Higher estimates of δ (in ms) therefore suggest longer periods of uncertainty about the lexical status of each letter string, and correspondingly, longer periods of plain visual information uptake. The composite measure γ × β/(1 + γ) is an estimate of what we previously called *home-in time*, where perceivers increasingly favor the target word over the distractor non-word as time progresses. More precisely, γ × β /(1 + γ) determines the time that elapses between the peak location (δ) and the point in time where cumulative average proportions of fixations on the distractor have fallen back to exactly half-amplitude level. Thus, a higher estimate of γ × β /(1 + γ) (in ms) indicates that it takes perceivers proportionally longer to disengage themselves from the distractor non-word (and to home-in on the target word, respectively) after having overcome the initial uncertainty period. The third measure of interest is the peak amplitude (λ), i.e., the estimate of the overall height of the PD(*t*) curve. While the previous two measures are primarily concerned with processing *speed*, this latter measure relates to the overall *amount of errors* made during the 3-s trial period.

## Results

**Figure 4:**
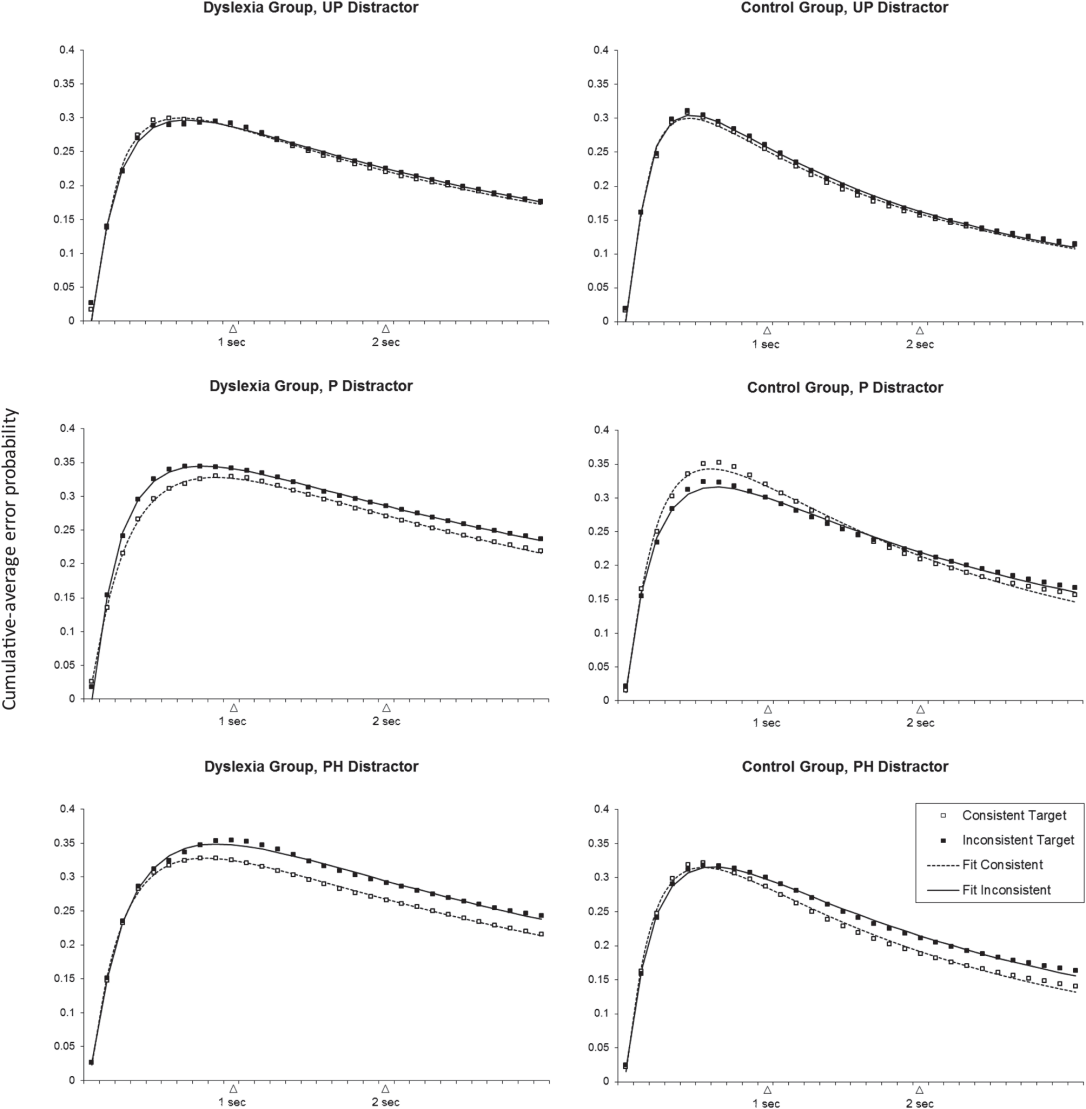
**Cumulative-average error probabilities over time (10 Hz sampling rate) in each condition**. Panels at the left show data for the Dyslexia group, panels at the right for the Control group. From top to bottom: UP, unpronounceable; P, pronounceable; PH, pseudohomophone distractor condition. Open symbols represent observed data for the rhyme-consistent target condition, filled symbols for the rhyme-inconsistent target condition. Log-normal peak model fits are indicated by dashed and solid lines, respectively.

Figure 4 shows mean cumulative-average probabilities of fixations on the distractor non-word over time, separately for each participant group and condition. The dotted and solid lines in the figure indicate the best fit of the grand average data per condition using (Equation 1). The corresponding parameter estimates, as well as average parameter estimates derived from fitting (Equation 1) to individual participant × condition data sets, are shown in Table 2; note that parameter estimates from the grand average fit (first value per cell in Table 2) are mostly within one standard error of the means across the individual participant models (second and third value per cell in Table 2).

**Table 2:** **Parameter estimates for the grand average data per condition (first value per cell) and for individual participant data per condition [second and third value per cell, showing means, and standard errors (in brackets) across subjects]; the *amplitude* parameter (λ) is measured in probability units; the parameters *peak location* (“*initial uncertainty time*,” δ), *width* (β) and the compound measure “*home-in time*” (γ × β/(1 + γ)) are in millisecond units**.

**Group**	**Distractor**	**Rhyme-inconsistent target**					**Rhyme-consistent target**				
		**λ**	**δ**	**β**	**γ**	**γ × β/(1 + γ)**	**λ**	**δ**	**β**	**γ**	**γ × β/(1 + γ)**
Dyslexic	UP	0.297	714	3566	6.04	3059	0.300	679	3411	6.27	2942
		0.305 (0.014)	744 (64)	3630 (418)	5.96 (0.55)	3099 (375)	0.311 (0.012)	725 (61)	3385 (390)	6.14 (0.60)	2881 (340)
	P	0.345	816	4566	6.62	3967	0.328	896	4046	5.06	3378
		0.358 (0.013)	929 (108)	5273 (938)	6.22 (0.42)	4574 (844)	0.336 (0.016)	878 (60)	4143 (364)	5.75 (0.54)	3511 (344)
	PH	0.349	907	4347	5.36	3664	0.328	831	4088	5.58	3467
		0.354 (0.016)	915 (60)	4768 (516)	5.92 (0.54)	4083 (477)	0.335 (0.014)	797 (62)	4405 (444)	7.87 (1.47)	3834 (442)
Control	UP	0.305	520	1957	5.04	1633	0.300	512	1945	5.16	1629
		0.309 (0.013)	534 (58)	1943 (381)	4.91 (0.50)	1608 (342)	0.301 (0.011)	524 (55)	1950 (356)	4.97 (0.55)	1618 (311)
	P	0.316	680	2865	4.96	2384	0.343	646	2360	4.38	1921
		0.321 (0.012)	682 (99)	2952 (857)	5.21 (0.38)	2477 (771)	0.346 (0.015)	649 (55)	2342 (332)	4.40 (0.49)	1904 (314)
	PH	0.316	660	2756	4.92	2290	0.315	599	2315	4.67	1907
		0.321 (0.014)	664 (55)	2733 (471)	5.00 (0.38)	2271 (436)	0.322 (0.013)	602 (55)	2288 (405)	4.77 (1.34)	1886 (404)

Symmetry (γ) has no unit of measurement as it indexes a ratio. UP, unpronounceable; P, pronounceable; PH, pseudohomophone.

Across participant models, the three measures of interest (overall error rate, initial uncertainty time, and home-in time) were statistically analyzed using Three-Way mixed design ANOVAs with group (dyslexic vs. control) as a between-subjects factor and target (inconsistent vs. consistent) and distractor type (UP, P, PH) as within-subjects factors. Since the speed-related measures (initial uncertainty time and home-in time) both showed a strong positive skew in their raw distributions, they were log-transformed to better comply with the parametric assumptions of ANOVA. Table 3 summarizes the results.

**Table 3:** **ANOVA results for the three measures of interest: overall error rate [*λ*], log initial uncertainty time [*ln*(*δ*)], and log home-in time [*ln*(γ × β/(1 +γ))]**.

**Measure**	**Effect**	***df***	***F***	***p*η^2^**
λ	Group	1.20	0.63	0.03
	Target	1.20	0.78	0.04
	Distractor	2.40	19.04^**^	0.49
	Group × target	1.20	7.36^*^	0.27
	Group × distractor	2.40	1.64	0.08
	Target × distractor	2.39	0.63	0.03
*ln(δ)*	Group	1.20	9.33^**^	0.32
	Target	1.20	7.37^*^	0.27
	Distractor	2.35	38.68^**^	0.66
	Group × target	1.20	0.10	0.01
	Group × distractor	2.35	0.24	0.01
	Target × distractor	2.34	1.78	0.08
*ln*(*γ* × β/(1 + *γ*))	Group	1.20	16.24^**^	0.45
	Target	1.20	10.78^**^	0.35
	Distractor	2.31	26.95^**^	0.57
	Group × target	1.20	0.53	0.03
	Group × distractor	2.31	0.20	0.02
	Target × distractor	2.40	2.01	0.09
	Group × target × distractor	2.40	1.05	0.05

Group was a between-subjects factor, and Target and Distractor were within-subjects factors. Where appropriate, degrees of freedom (rounded to nearest integer in the table) were adjusted using the Greenhouse-Geisser correction. The rightmost column reports partial eta-square (p^η^^η2^), a standardized measure of effect size. Asterisks indicate significant effects (

* p < 0.05;

** p < 0.01).

### Overall error rate: λ

**Figure 5:**
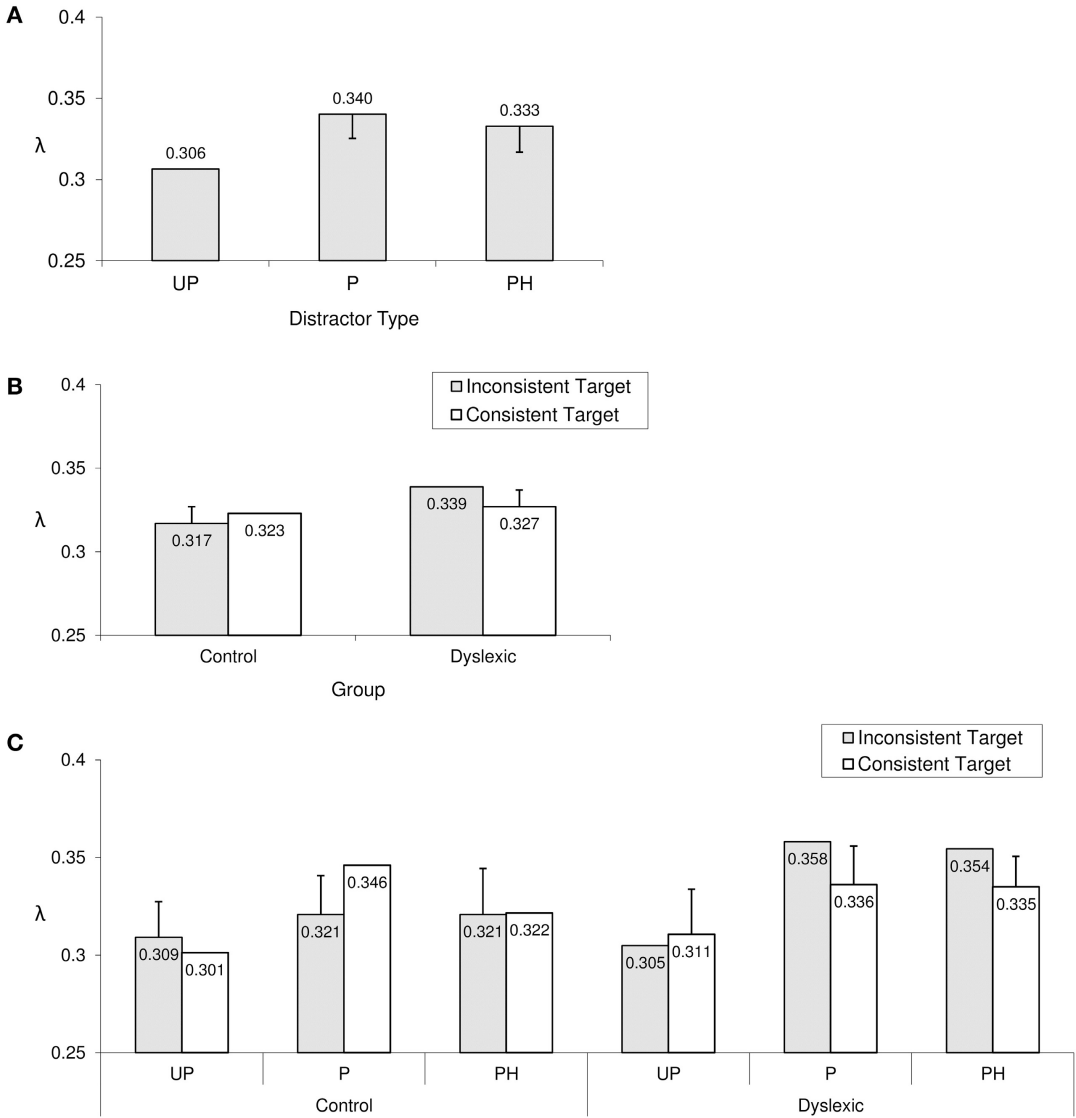
**Significant effects in overall error rate (λ). (A)** The main effect of distractor type; **(B)** the group × target type interaction; **(C)** the group × target × distractor interaction. Error bars represent Sidak-adjusted 95% confidence half-widths for **(A)** contrasts with the UP distractor condition, **(B)** the target type simple effect per group, and **(C)** the target type simple effect in each group × distractor type combination. Significant differences must exceed those half-widths. UP, unpronounceable; P, pronounceable; PH, pseudohomophone.

Regardless of participant group and type of target word, different types of non-word distractors led to significantly different overall error rates, as reflected in a significant main effect of distractor type on the λ estimates. As shown in Figure 5A, there was no difference between P and PH distractors. However, both P and PH distractors were associated with significantly higher overall error rates in comparison to UP distractors. The UP < P pattern was carried by 9/10 dyslexics and 11/12 controls. The UP < PH pattern was observed in 10/10 dyslexics and 10/12 controls.

The two-way interaction between group and target type was also significant. As can be seen in Figure 5B, rhyme-inconsistent target words led to significantly higher overall error rates only in the dyslexic group, where this pattern (consistent<inconsistent) showed up in 7/10 participants. In the control group, by contrast, no such simple effect of rhyme-consistency was observed (only 5/12 control participants displayed lower overall error rates when the target word was rhyme-consistent rather than rhyme-inconsistent).

The three-way interaction between group, target type and distractor type was also significant, indicating that the group by target type interaction differed depending on distractor type. As can be seen in Figure 5C, the previously observed rhyme-consistency contrast for the dyslexic group (lower overall error rate with consistent rather than inconsistent target words) was significant only with P and PH distractors, each time showing up in 8/10 dyslexic participants. In contrast, the control group displayed the *opposite* pattern in combination with P distractors, i.e., rhyme-consistent target words led to *higher* overall error rates than rhyme-inconsistent target words in the P distractor condition (an effect that was carried by 10/12 control participants). This pattern for the control group is rather unexpected and deserves further examination. Indeed, Figure 4 suggests that in this particular factor combination (Control Group, P distractor condition), the higher peak error rate for consistent target words is later compensated by a faster home-in time on those consistent target words (note that in this factor combination, the curves for the consistent and inconsistent target word condition intersect each other at around 1700 ms from stimulus onset). Hence, in this particular case, the peak amplitude λ may not be as indicative of overall error rate as in the other conditions.

### Initial uncertainty time: ln(δ)

**Figure 6:**
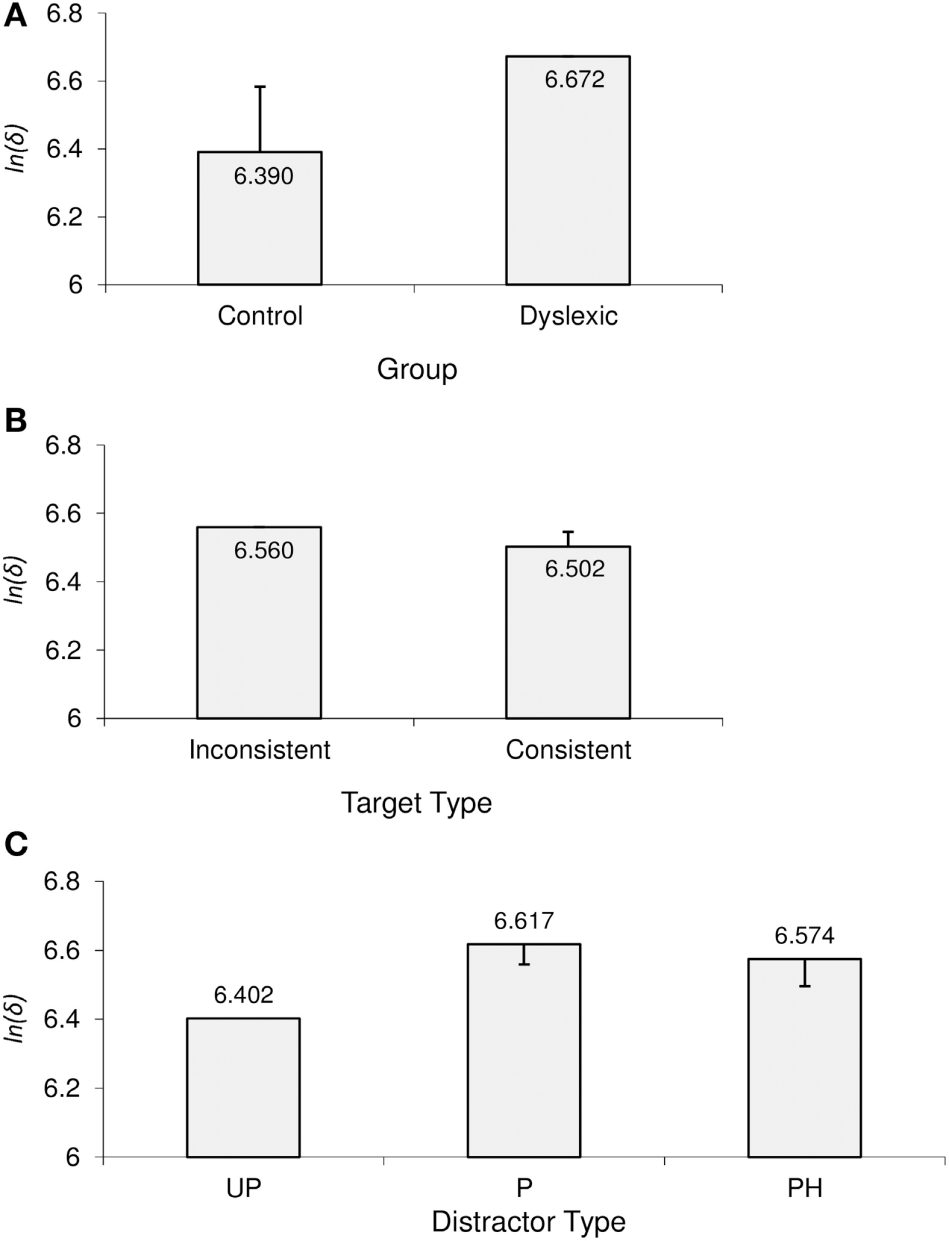
**Significant effects in initial uncertainty time [ln(δ)]**. **(A)** Main effect of group; **(B)** main effect of target type; **(C)** main effect of distractor type. Error bars represent Sidak-adjusted 95% confidence half-widths for **(A)** the group contrast, **(B)** the target type contrast, and **(C)** contrasts with the UP distractor condition. Significant differences must exceed those half-widths. UP, unpronounceable; P, pronounceable; PH, pseudohomophone.

As can be seen in Figure 6A, the main effect of participant group on initial uncertainty time was due to dyslexic participants exhibiting significantly longer initial uncertainty times than controls. For a more qualitative analysis, non-overlapping groups would show median ranks of 17 (dyslexics) and 6 (controls), i.e., the dyslexic group would be uniformly slower without the quickest dyslexic being faster than the slowest control participant. Observed median ranks of 15.5 (dyslexics) and 6.5 (controls) indicated that dyslexic participants were indeed near-uniformly slower than controls.

There was also a significant main effect of target type. As can be seen in Figure 6B, rhyme-inconsistent target words led to longer initial uncertainty times compared to rhyme-consistent target words. Qualitatively, this was true for 8/10 dyslexic and 10/12 control participants.

Finally, there was a main effect of distractor type on initial uncertainty time. Consistent with the previously observed pattern in overall error rate, Figure 6C shows that P and PH distractors did not differ in terms of associated initial uncertainty time. However, both P and PH distractors were associated with significantly increased initial uncertainty times compared to the UP distractor condition; the UP < P contrast showed up in the models for all participants, and the UP < PH contrast in all but one dyslexic participant's model.

### Home-in time: ln (γ × β/(1 + γ))

**Figure 7:**
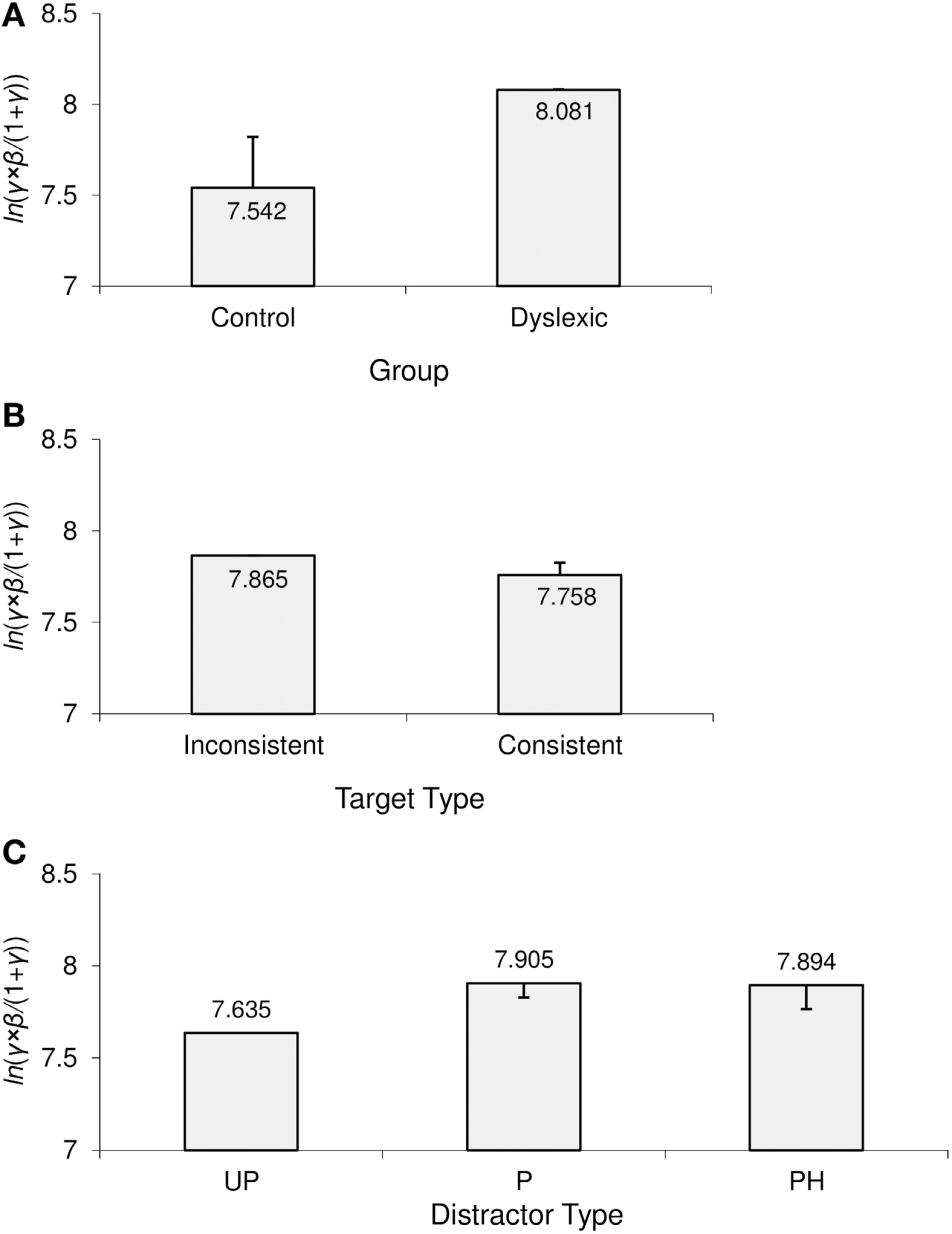
**Significant effects in home-in time [ln(γ × β/(1 + γ))]**. **(A)** Main effect of group; **(B)** main effect of target type; **(C)** main effect of distractor type. Error bars represent Sidak-adjusted 95% confidence half-widths for **(A)** the group contrast, **(B)** the target type contrast, and **(C)** contrasts with the UP distractor condition. Significant differences must exceed those half-widths. UP, unpronounceable; P, pronounceable; PH, pseudohomophone.

Overall, the home-in time measure revealed almost identical effect patterns to those observed in initial uncertainty time. As shown in Figure 7A, compared to the control group, dyslexic participants took significantly longer to home-in on the target (i.e., to reach half-amplitude level in fixations on the distractor). In terms of group overlap, the median rank of the dyslexic group was 16.5, i.e., very close to the ideal 17; the median rank of the control group was 6.5, i.e., just above the ideal of 6. This suggests that dyslexics were nearly uniformly slower than controls in homing-in on the target.

The main effect of target type was also significant. As can be seen in Figure 7B, it took participants longer to home in on rhyme-inconsistent target words than on rhyme-consistent target words. This was reflected in the average data of 7/10 dyslexics and of 10/12 controls.

Finally, the main effect of distractor type was significant due to the pattern illustrated in Figure 7C. In line with the results from the previous two measures, there was no significant home-in time difference between the P and PH distractor conditions. However, both the P and PH distractor conditions exhibited increased home-in times compared to the UP distractor condition. The UP < P contrast showed up in all participants and the UP < PH contrast in all but one dyslexic and one control participant.

## Discussion

The present study used a novel two-alternative forced choice LDT (based on eye-tracking) in order to compare word-recognition performance in normal and dyslexic adult readers. It was found that dyslexic readers—relative to controls—were generally less accurate (i.e., more likely to make errors) in recognizing rhyme-inconsistent target words as opposed to rhyme-consistent target words, even though they were given plenty of time to perform the task. This finding was in addition to a general slowdown in the speed of access to rhyme-inconsistent words, which was observed in dyslexic and control participants alike. Indeed, the two speed-related measures (initial uncertainty time and home-in time) both registered a group-*independent* rhyme-consistency effect whereby rhyme-inconsistent target words were generally recognized more slowly than rhyme-consistent target words. Thus, while rhyme-consistency of the target word generally affected processing speed, it was only the dyslexic readers who also showed a corresponding effect in overall response accuracy. The latter could suggest a *representational* deficit for rhyme-inconsistent words in adult dyslexic readers, above and beyond a group-independent deficit in the speed of access to rhyme-inconsistent words.

In line with previous findings, we also found that dyslexic readers were generally slower than age- and gender-matched controls, both in terms of initial information uptake and in terms of homing-in on the target word. Surprisingly, the present study found no evidence for a *pseudohomophone effect*, which had been documented in some of the earlier research quoted in the introduction. In the present task, there were no differences in terms of speed or accuracy between rejecting P or PH distractors, in spite of the fact that all three measures (overall error rate, initial uncertainty time, and home-in time) indicated that UP distractors were generally easier to reject than either of the other two types of distractor non-words. These latter differences were independent of participant group. In what follows, we will discuss our findings in more detail.

### Rhyme-consistency

The rhyme-consistency findings were partly surprising. In contrast to Lacruz and Folk (2004); Ziegler et al. (2008); Stone et al. (1997), our group of normal readers did not exhibit more accurate responses for rhyme-consistent than for rhyme-inconsistent target words. It could be argued that this effect only emerges with appropriate non-word distractors in the stimulus list. However, Gibbs and Van Orden (1998) showed that rhyme-consistency effects do emerge when at least 30% of the non-words are PH. This suggests that the absence of a rhyme-consistency effect in the accuracy of the present sample of normal readers is not due to the characteristics of our set of non-word distractors. Given that the expected rhyme-consistency effect was present in speed-related measures, the present findings instead suggest that those previously reported consistency effects on lexical decision accuracy in normal readers could be due to a speed-accuracy trade-off whereby participants made more errors for a gain in speed when responding to rhyme-inconsistent words. Recall that in contrast to the present study, those previous findings were based on conventional reaction time tasks where participants could trade off speed against accuracy.

In comparison, our dyslexic group of participants did show an accuracy impairment in judging rhyme-inconsistent words (particularly in combination with P and PH distractors) which lasted over the whole three-second trial period. This suggests that dyslexic participants might suffer from a speed-of-access deficit *as well as* a representational deficit for rhyme-inconsistent words.

In terms of Coltheart et al.'s (2001) DRC model, this argues for a dyslexic deficit in the non-lexical route of processing, as these inconsistent words are likely to be more difficult to process for the grapheme-phoneme rule system compared to consistent words^6^. This difficulty is also apparent in the greater processing time needed by non-dyslexic readers who appear to have (at least partially) relied on the non-lexical route in order to solve the task. However, while control participants were able to overcome this difficulty with sufficient time, dyslexic readers were generally less likely to recognize rhyme-inconsistent stimuli as words, particularly when these stimuli were combined with P or PH distractors.

It should be noted that the consistency norms applied in this study (Ziegler et al., 1997) are based on American dictionary pronunciations whose applicability to the British language background of our participants may be questionable. However, feedforward and feedback consistency manipulations (based on the same norms) have previously been found to affect British children's reading and spelling (Davies and Weekes, 2005). This suggests that Ziegler et al.'s (1997) norms are still very useful for studies on native speakers of British English. More importantly, pronunciation differences between American and British English should actually have worked *against* finding any effects of the consistency manipulation in our study. Therefore, finding consistency effects in our sample of participants can only speak for the strength of the manipulation, even though a replication on the basis of more “regional” norms (once available) would be desirable in future research.

### Distractor-related effects

Compared to P and PH non-word distractors, UP non-word-distractors were generally quite easy to dismiss as non-words, and this was the case both for dyslexic and for normal readers. This aspect of our results was perhaps the least surprising because UP non-words are not only phonologically infelicitous, but can also be rejected based on a pre-lexical analysis which identifies illegal graphemes.

Unexpectedly, however, no pseudohomophone effect was found in the present study, neither in dyslexic nor in normal readers. That is, there was no indication of delayed (or less accurate) word recognition when the target word was combined with a PH distractor as opposed to a “standard” pronounceable non-word distractor (P).

While we expected the absence of a PH effect for dyslexic readers (in accordance with the phonological deficit hypothesis, e.g., Vellutino et al., 2004; Swanson and Hsieh, 2009), its absence in unimpaired adult readers warrants closer examination, as it might call the pervasiveness of such effects (Frost, 1998) into question. In terms of accuracy, the absence of a PH effect in our study is actually in line with Atchley et al.'s (2003) findings on Dutch children. These authors originally suggested that the absence of a PH effect in response accuracy could have been due to the speed requirements of their task—a conjecture that is not as easily applicable to the paradigm used in the present experiment.

Given that the present task has not been applied to the processing of PH before, it could be argued that our method was not sensitive enough to find this effect in healthy adult readers. However, none of our three measures of interest showed even a non-significant trend in the predicted direction. Moreover, the methodology was sensitive enough to register processing differences between UP non-words on the one hand and pronounceable (P and PH) non-words on the other, showing significant contrasts with the UP condition in all three measures of interest. Thus, the absent PH effect is unlikely to be an artifact of task sensitivity.

Could orthographic neighborhood characteristics of our stimuli have suppressed the emergence of a PH effect? Indeed, computational simulations by Coltheart et al. (2001) and by Seidenberg and McClelland (1989) suggest that PH which are orthographic neighbors to their base words should show stronger effects than PH which are not. In the present study, around 43% of the PH distractors were orthographic neighbors to their base words. Again, this would predict at least a non-significant trend in the direction of a PH effect. Instead, our experiment registered non-significant trends in the opposite direction in both dyslexic and normal adult readers.

Finally, there may also have been problems with a subset of the stimuli we have used (see Supplementary Materials). As one reviewer noted, some of our PH distractors were arguably very similar, but not identical in pronunciation to their corresponding base words, e.g., *bensh* - *bench*. Other examples included unusual letter combinations (e.g., *tekst*) which may have been exploited by participants even though we controlled for bigram frequency. While keeping such limitations in mind, we tentatively conclude that the absence of a PH effect in healthy adult readers might question the role of the phonological lexicon in making lexical decisions on written stimuli. Further research, perhaps based on more established pseudohomophone examples from the existing literature, is definitely needed in order to conclusively resolve the issue.

### Implications for current theories of word recognition and dyslexia

The findings obtained from the present study have implications for theories of word recognition and for theories of dyslexia. First of all, the absence of a PH effect and the presence of a “pronounceability” effect in non-word recognition could imply that lexical decisions are not dominated by whole-word phonological representations. The rhyme-consistency effects provide further support for this conclusion. Our findings suggest instead that different levels of sublexical representation are used by readers to distinguish words from non-words: the grapheme/phoneme level (orthographic awareness of rules and conventions and phonological decoding) and the rhyme level. This can be taken as evidence for the importance of the non-lexical route of processing in Coltheart et al.'s (2001) DRC model. Apparently, even when the correct functioning of this route is impaired—as in dyslexia—the reader still relies on it, as seen in the effects of our rhyme-consistency manipulation.

In terms of dyslexia theories, the findings presented here add to the literature suggesting a general speed deficit in dyslexic readers (e.g., Stoodley and Stein, 2006; Laasonen et al., 2009; Swanson and Hsieh, 2009). Thus, Wolf and Bowers's (1999) suggestion of a speed deficit as one core impairment of dyslexia receives support. From our data, it seems difficult to pinpoint the precise location of this deficit in the reading architecture, as dyslexics appeared uniformly slower than control participants across all conditions and measures. A phonological core deficit (Vellutino et al., 2004) is also supported given the dyslexic problem of accurately recognizing rhyme-inconsistent words that we observed here.

Going beyond the theories which motivated the design of this study, it is worth pointing out that dyslexia is unlikely to be a uniform disorder. For example, Castles and Coltheart (1993) have differentiated between surface and phonological dyslexics. Our dyslexic sample likely included mixed types only, bearing in mind that the LADS dyslexia screener targeted both lexical access (word recognition) and phonological encoding (word construction) and that a mixed profile of dyslexia is typically most prevalent (e.g., Castles and Coltheart, 1993; Bergmann and Wimmer, 2008). Also, our dyslexic participants appeared relatively homogenous in their performance, which might argue against a categorization of its members into dyslexic subtypes. Thus, our results speak to developmental dyslexia in general rather than any particular subtype of dyslexia.

However, could other deficits in dyslexic readers have influenced our results? Bosse et al. (2007) suggest that many dyslexics suffer from a small attentional window which prevents them from processing words as a whole. Such a proposal could indeed explain dyslexic deficits in early processing measures such as *initial uncertainty time* in the present study. However, it remains unclear whether such early attentional problems could also explain later effects in *home-in time* or indeed the observed accuracy deficit in identifying rhyme-inconsistent words in dyslexic readers.

Conversely, some authors have proposed a generally “sluggish” attentional system in dyslexic readers (Hari and Renvall, 2001), which might explain why in our task dyslexics took longer to *home-in* on the target than control participants (dyslexics might need more time to disengage their attention from the distractor). However, this would not account for why *initial uncertainty time* (the early time period where participants could not reliably discriminate between target and distractor) was also prolonged for dyslexic readers. In sum, a phonological deficit in combination with a dyslexic processing speed deficit appears like the most parsimonious explanation for our findings.

### Speed-vs.-accuracy trade-offs

The potential methodological advantages of the current task should not be underestimated. As opposed to classical LDTs based on single button responses per stimulus, we were able to distinguish differences in processing speed from differences in processing accuracy. For example, two effects were indicative of speed-of-access differences without accompanying accuracy differences: One was the rhyme-consistency effect in control participants and the other was the general speed impairment in dyslexic readers. All other speed-related findings were mirrored in accuracy-related results. As discussed earlier, such a differentiation between speed- and accuracy-related effects is very difficult to achieve with classical LDTs where the generative process behind the data remains ambiguous. For example, the rhyme-consistency effect in control participants was only detected in speed-related measures in our study, but in a classical lexical decision experiment, it may well show up in response accuracy if participants have set their response criteria accordingly^7^.

In comparison to the response signal paradigm (a well-attested method to get around the problem of speed-accuracy tradeoffs in responding), our new method has the advantage of being more easily applicable, as it does not require any training of participants. Moreover, exclusion of trials due to poor cue-reaction performance is largely unnecessary. Another approach to avoiding speed-accuracy trade-offs is computational modeling of responses in classical lexical decision experiments (e.g., Zeguers et al., 2011). However, while this approach elegantly combines reaction time and accuracy measures, possible inferences are limited by (i) the availability of enough error trials in all experimental cells, (ii) assumptions about the constancy of model parameters across experimental cells, and (iii) the parameters included in the model. For example, with the approach taken by Zeguers et al. (2011) it would not be possible to distinguish between a speed- and an accuracy-related effect as done in the present study.

In sum, it still remains to be seen whether the current method is a suitable candidate to complement or even replace other approaches to overcoming the speed-accuracy trade-off under some circumstances. Given its relative simplicity, and the growing availability of eye-tracking facilities, we believe that it holds some promise in these respects.

## Conclusions

Using a novel two-alternative forced choice LDT which avoids SAT of classical lexical decision paradigms, the present study is the first to demonstrate a selective impairment for dyslexic readers in responding to rhyme-inconsistent words. This suggests that dyslexics possess deficient phonological representations for such words. Furthermore, we found that dyslexic readers appear to suffer from a general word-recognition speed deficit and that rhyme-inconsistent words were processed more slowly by both dyslexic and normal readers. Finally, the present study might call into question the pervasiveness of the PH effect which was neither found in dyslexic nor in control participants even though the method was sensitive enough to find effects of pronounceability. Thus, we found that in the present task, lexical decisions were mostly affected by sub-lexical representations.

## Author contributions

Richard Kunert and Christoph Scheepers designed the study and wrote the manuscript. Richard Kunert developed materials and collected the data. Christoph Scheepers analyzed the data.

### Conflict of interest statement

The authors declare that the research was conducted in the absence of any commercial or financial relationships that could be construed as a potential conflict of interest.

## Footnotes

^1^Note that other tasks which are commonly used to investigate dyslexic impairments face the same issue. A non-exhaustive list includes linguistic tasks such as word identification and word attack (reading aloud), rapid automatized naming, and letter identification (*cf*. Vellutino et al., 1996). Moreover, sensory tasks such as visual (e.g., random dot motion perception, *cf*. Stein, 2003) and auditory tasks (e.g., rapid perception test, *cf*. Tallal, 1980) face the same challenge of distinguishing between processing speed and accuracy.

^2^Note that Coltheart et al.'s (2001) DRC model is only concerned with reading, and therefore makes no predictions concerning feed *back* consistency. However, because there is a growing empirical literature on the importance of feedback consistency for reading in general (Davies and Weekes, 2005) and for word processing in particular (Stone et al., 1997; Bosman et al., 2006) we decided to consider this factor as well in our manipulations. See also Stone et al. (1997); Davies and Weekes (2005) for theoretical accounts of feedback consistency effects in reading.

^3^ Indeed, research comparing classical RTs with results from the response signal paradigm has shown that classical RT responses typically undercut the asymptotic accuracy level, as illustrated in Figure 1.

^4^ There were 15 such pair-wise comparisons. Since the focus was on establishing *non-significant* differences, no family-wise error correction was applied to maximize power.

^5^Note that cumulative-average smoothing is relatively simple and straightforward, contrasting with other smoothing techniques that require the specification of tuning parameters (kernel-size etc.) to control the extent of smoothing.

^6^Another interpretation would be a mismatch resolution problem: Inconsistent words might activate different entries in the lexicon, based on a lexical reading, and a non-lexical reading. Resolving the conflict between these two entries might require extra processing time. While this could account for consistency effects in processing speed, it does not account for the *accuracy* deficits we observed in our sample of dyslexic readers. We would like to thank an anonymous reviewer for this suggestion.

^7^It should be noted that there are other differences between the current task and classical lexical decision tasks. While in the latter participants typically judge one letter string at a time, the current task requires participants to choose between two simultaneously presented stimuli, adding a competitive element to the lexical decision process.
