# Supplementary material for: Speed and accuracy of dyslexic versus typical word recognition: an eye-movement investigation
Source: Front Psychol. 2014 Oct 9;5:1129. doi: 10.3389/fpsyg.2014.01129 (PMC4191135; doi:10.3389/fpsyg.2014.01129)
Supplement: Supplementary file 1 [file DataSheet1.DOCX]

**Supplementary Material.** Stimuli.

| inconsistent – UP | inconsistent – P | inconsistent – PH | consistent – UP | consistent – P | consistent - PH |
| --- | --- | --- | --- | --- | --- |
| sphere - clpter  screen - llells  chrome - ptrefr  chough - vitnss  blare - nhtin  great - rernr  pleat - dpler  flare - lencm  green - hzner  stove - rscat  beard - honkh  gross - inxcg  prove - sishq  worth - slitc  youth - bbesh  close - pstaz  tooth - ghsan  mould - wcmar  whose - wrdio  glove - dparp  broad - vatsj  gnome - mneez  snood - molnw  squad - gmcig  care - wter  mare - rder  neat - esss  hone - ntim  hare - psti  meat - ontc  heat - snti  pear - dcin  wand - enng  cord - innn  cork - icng  rave - litc  ward - cces  ruse - mrou  warm - qrte  deaf - ptro  tome - fmti  love - enlt  crow - vteo  dork - katb  fork - bllu  nave - fesw  show - cfes  pave - eskg  move - aljr  wool - orwm  whom - hrun  foot - eaof  gild - liqq  jowl - gwsa  ton - hti  pro - kte  tow - sso  gas - wca  put - fma  key - htu  ski - ibt | breast - tength  grease - realth  sheath - mierce  cruise - trarch  slough - straft  squash - trodge  height - progue  stein - trong  caste - pinge  share - soint  glare - crang  clone - rount  plant - loist  stork - lound  geese - trelp  wheat - tedge  trove - roice  grave - zench  brave - sarsh  gaunt - troil  phase - strux  jaunt - rield  blood - setch  brood - dilth  swan - teps  bough - pruck  sough - mague  sword - rymph  knave - quafe  bone - kler  fare - tren  wont - stig  peat - gren  zone - steo  bear - rell  coup - pesh  keen - jang  tour - blex  pram - trid  wart - nelf  twat - frep  raid - arge  four - rask  cave - teft  foul - rilk  pork - gick  home - zald  dose - frib  limb - simp  worm - dact  dome - susk  cowl - hept  tomb - mibe  wave - toft  slow - pafe  food - sobe  snow - wapt  womb - wext  son - lem  don - hab  yea - wex | throne - balled  wreath - trensh  crease - cintch  square - pintch  trough - fierse  bruise - cloath  stone - intch  grant - tecst  steak - tensh  chant - liesh  spare - tekst  slant - balld  stour - mincs  sheen - coche  stave - artch  taunt - ledje  cache - cravt  chord - tchit  haunt - helth  rough - proab  farce - shelv  ghoul - promt  queen - selph  grove - gravt  tough - rapht  slave - limph  shave - moyst  clove - prowb  plaid - phrag  dough - coacs  fjord - flask  flood - sedje  swamp - poynt  mere - nell  here - desc  cone - slic  tone - knel  lone - cick  seat - casc  bare - tasc  ware - scit  this - roab  what - tusc  thou - cowx  lord - ravt  cove - rowb  mead - loab  warp - laps  comb - milc  your - chik  rood - lofe  maid - ploi  shoe - coyl  soot - coyn  mood - kusp  fowl - cilk  wan - rit  sew - sel  low - wen  mot - hic  bow - cel | string - blnere  stench - dtrerh  stetch - wtrite  twinge - ddbint  strife - lerbwk  strobe - therhq  crotch - ttlase  shrimp - endfrd  fridge - necltb  tinge - nterv  thing - scter  hinge - htest  finch - lcont  wrong - nentv  winch - whner  joint - erngm  fresh - rinhz  bench - onntl  crisp - ntlef  ounce - patrl  brick - frrel  crust - sedll  torch - ermgm  thick - krred  coach - tsjin  barge - fllip  sedge - pmany  vetch - wtetb  ridge - ricbm  lymph - kgmes  wedge - gwrir  theft - qarhq  ring - nter  ding - qrer  king - erll  inch - ntan  minx - hmin  fang - insj  stag - ttiv  grit - llig  mien - athz  sick - hrec  arch - wcti  tact - onnj  brig - rsly  left - qenn  help - alhn  milk - rxli  bulk - dpog  club - hryp  gift - emdc  sex - rdi  elf - upr  pig - vdi  big - idr  apt - ifr  fox - nwa  bug - umr  mug - hqu  jug - mgi  job - jpu | strength - sprierce  spring - stoint  starch - sealth  wealth - scarch  choice - stotch  broach - kletch  bridge - shilth  shrift - crogue  brogue - sproys  fling - rinch  prong - stide  wrong - drang  groin - jinge  moist - gench  snell - tarve  march - toost  large - narge  chick - stafe  stump - boice  tribe - jarsh  probe - grilk  voice - prook  quick - plext  bribe - soach  craft - lompt  truck - shoft  judge - zodge  coin - lerc  long - roin  tang - tisp  loin - vist  glen - noin  pang - rish  lien - wang  dang - winx  rick - hesh  rust - telf  lust - cald  dust - pust  cell - pell  just - frit  wish - hisp  bell - blid  stub - orch  crag - seld  text - trob  self - gick  wick - rimp  grid - delp  lift - dact  fact - laft  luck - gask  fife - gept  duke - jibe  men - cen  rag - lep  bag - hig  mob - dag  sub - wem  pub - yex  joy - zib | strong - stensh  health - coatch  sphinx - martch  scotch - bardge  brunch - scetch  scribe - scetsh  thrift - philth  sketch - shapht  sling - bensh  tench - tinje  point - phang  pinch - herts  mount - necst  wench - hinje  knell - krisp  slick - wensh  shell - nekst  noise - chois  porch - roche  roach - scoch  sprig - djust  shelf - welth  bunch - lepht  hedge - trakt  hunch - seaje  filth - ligue  graft - celph  draft - ridje  ping - lenz  tick - wive  wren - bric  gong - rivt  bang - snel  gang - elce  mesh - shel  dish - quic  gust - temt  hell - vois  fish - sics  writ - secs  prig - masc  coil - livt  colt - morg  rapt - krag  pact - cect  edge - rusc  silk - coxe  soil - silc  wife - lymf  drug - mosc  tag - bel  hem - wel  oil - cex  fig - els  toy - elc  six - toi  cup - sik  tug - boi  boy - nob  box - soi  rug - luc |
